# Therapeutic efficacy of chloroquine for treatment of *Plasmodium vivax* malaria cases in Guragae zone southern Central Ethiopia

**DOI:** 10.1186/s12879-019-4055-7

**Published:** 2019-05-14

**Authors:** Teha Shumbej, Abdulewhab Jemal, Abdulsemed Worku, Fitsum Bekele, Habtamu Weldesenbet

**Affiliations:** 1 Department of Medical Laboratory Sciences, College of Medicine and Health Sciences, Wolkite University, Wolkite, Ethiopia; 2 Department of Medicine, College of Medicine and Health Sciences, Wolkite University, Wolkite, Ethiopia

**Keywords:** *Plasmodium vivax*, Therapeutic efficacy, Guragae zone, Ethiopia

## Abstract

**Background:**

Malaria continues to be a public health problem and important cause of morbidity and mortality in Ethiopia. Due to continuous interventions to combat malaria in endemic regions, a decline in malaria related deaths and morbidity has been registered. These gains, however, are threatened with the emergency of antimalarial drugs resistant strains of plasmodium parasites. This study aimed to determine therapeutic efficacy of chloroquine for treatment of *Plasmodium vivax* malaria cases in Guragae zone, southern central Ethiopia.

**Methods:**

A one arm prospective study with recurrence of parasitaemia and clinical conditions of patients were evaluated on days 0, 1, 2, 3, 7, 14, and 28. Patients with *Plasmodium vivax* malaria mono infection and eligible for study inclusion criteria were recruited. SPSS-21 used for data analysis and management. Kaplan-Meier survival probability analysis was estimated. Mean geometric parasitaemia and average haemoglobin concentration were calculated.

**Results:**

Among 87 total recruited subjects, 81 of them completed the 28 days follow up. More than half of (57.5%) the study participants had a history of fever and 42.5% of them had fever at the time of enrollment. The mean body temperature on day of recruitment was 38.2 °C and 36.8 °C on day 28. Geometric mean parasitaemia calculated on day of enrollment was 2270 parasites/μl of blood. Recurrence of parasitaemia was registered from two subjects during entire follow up. The mean haemoglobin concentration of study participants on day of enrolment was 11.8 g/ dl and 13.8 g/dl on day 28.

**Conclusion:**

This study registered a high chloroquine efficacy rate among the study participants. Therefore, chloroquine remains efficacious for the treatment of *Plasmodium vivax* malaria in the study area. However, there is a need to monitor chloroquine resistance by employing molecular tools for better evaluation of treatment outcome.

## Background

Malaria annually affects several hundred million people and malaria due to *Plasmodium vivax (P.vivax)* is the most important and a major public health problem [1]. Studies show that prevalence of *P.vivax* infection in east Africa, particularly in Ethiopia is higher [2]. Malaria is prevalent in 75% of Ethiopian land mass reporting 4 up to 5 million malaria cases annually. *P.vivax* distribution is mainly associated with altitude and rainfall. Vivax malaria accounts for up to 40% of malaria cases in Ethiopia [3]. According to 2016/2017 Gurage zone health department report, malaria ranks among the top ten diseases in the zone.

Prompting effective treatment is one of effective global strategies in fighting against *P.vivax* malaria as it benefit for both the patient and community. However, the effectiveness of such treatment strategy highly depends on the national policy of providing effective first line antimalarial drugs [2, 4]. Progress in eliminating *P.vivax* malaria need monitoring the anti vivax malarial drug for identifying emergence of multidrug resistant isolates [5]. Monitoring drug resistance is essential for timely changes to treatment policy, which should be initiated when the treatment failure rate in the country exceeds 10% [6, 7].

Despite the fact that emergence of chloroquine(CQ) resistant *P.vivax* isolates are reported from different parts of the world [8–10]. However, there are only few studies conducted on therapeutic efficacy of CQ for treatment of *P.vivax* in malaria endemic areas of Ethiopia [11, 12]. More studies are needed on the degree of CQ resistance of *P.vivax* in malaria endemic areas like Ethiopia to have clear picture of the country wide CQ resistance distribution. The present study gives updated information about the status of CQ resistance for the treatment of *P.vivax* in the study area.

## Methods

### Study period and area

The study was carried out from December, 2016 to May, 2017 in three selected health care centers located in Guragae zone, Southern Nations Nationalities, and People’s region. Guragae zone is located 155 k-meters south of Addis Ababa, the capital of Ethiopia. The zone has an annual rainfall of 800 to 1400 mm.

### Study design and population

A one-arm prospective study, with clinical and parasitological evaluation was done. 87 study subjects confirmed with *P.vivax* mono-infection on blood film examination and fulfilled the inclusion criteria were recruited. Sample size was calculated using single population proportion formula with expected 5% failure rate, a desired precision of 5 and 95% confidence interval (CI), and assuming an additional 20% for loss to follow-up [6]. Proportionate number of study participant in each selected health care center was determined and each study subject was recruited consecutively until the total sample size was attained.

### Inclusion criteria

Febrile patients (documented body temperature greater than 37.5 °C or having history of fever within the previous 48 h), who fulfilled other inclusion criteria and signed an informed consent was eligible for the study. Specifically, patients of aged > 6 months, microscopically confirmed *P. vivax* mono-infection, non-pregnant or non-breast-feeding women, permanently living within the health center catchment area (10 km radius) during the study period were recruited [6].

### Exclusion criteria

Study participants were excluded due to presence of other causes of fever at the time of screening, pregnancy, administration of any additional anti-malarial drugs during the study period, and subjects who vomit twice during drug administration [6].

### Patient enrolment, treatment and follow-up

Fixed schedule check-up visits were done on days 0, 1, 2, 3, 7, 14, 21 and 28. Corresponding clinical evaluation and laboratory examination were made. Baseline data on socio-demographic and clinical characteristics were recorded [6]. In areas endemic for *P.vivax* including in our study area, CQ is the first line drug [13]. Hence, patients were treated with a quality assured 25 mg/kg CQ (Addis Pharmaceuticals, Adigrat, batch number BN13079, date of expiration 06/2018), administered under direct observation for 3 consecutive days (10 mg/kg on days 0 and 1, and 5 mg/kg on day 2). Primaquine is an antimalarial drug given for radical cure, but it is not yet prescribed for vivax malaria in *P.vivax* endemic areas of Ethiopia. Subjects were checked for vomiting for 30 min after the drug ingestion. Blood smear microscopic examination was made in every scheduled visit. Moreover, haemoglobin concentration measurement was done on day 0, 28 and days of recurrence. Study participant failing to come to their scheduled appointment was traced to home assisted by the local guide. Study subjects were classified as having therapeutic failure and an adequate response [6]. Recurrence indicated when there is occurrence of *P.vivax* after the initial clearance of parasite from circulation. Subjects are considered to have cleared parasitaemia if there are at least two sequential negative smears and the day on which the first such negative smear is observed defined as the day of clearance [14].

### Drug quality analysis

The quality of CQ (Addis Pharmaceuticals, Adigrat, batch number BN13079, date of expiration 06/2018), used to treat all study participants was checked for its quality following the standard procedures before administration.

### Parasite detection

Thick and thin blood film microscopic examination was done to identify *P.vivax* mono infection. Asexual stages of *P.vivax* were counted against 200 white blood cells, assuming the average total white blood cell count of 8000/μl. Parasite density, expressed as the number of asexual parasites per μl of blood, was calculated by dividing the number of asexual parasites by the number of white blood cells counted and then multiplying it by an assumed white blood cell density (8000 per μl). Blood film examination was recorded as negative when examination of 1000 white blood cells revealed no asexual parasites. Parasite reduction ratio(PRR) was calculated using the formula PRR = Po/P2 (where Po is parasite count on day 0 and P2 parasite count on day 2) [6].

### Pregnancy test and haemoglobin determination

In pregnant females since Artesunate is recommended for treatment vivax malaria, female study participants aged 12 years and older were tested for pregnancy (WONDFO BIOTECH CO.LTD, China, batch number W00160542, date of expiration 06/2018). Haemoglobin concentration was also measured on day 0, on day of recurrence parasitaemia and on day 28 by using HemoCue Hb 301(Sweden) analyzer.

### Quality assurance

All positive and 10% of negative slides were picked randomly for quality control and re-examined by expert microscopist at Wolkite University parasitology Laboratory.

### Data analysis

SPSS version-21 (Chicago,USA) was used for data management and analysis. Kaplan-Meier survival probability analysis was used to calculate cumulative incidence of failure. Parasite reduction ratio, mean geometric parasite density and mean haemoglobin were calculated.

### Study variables and operational definition

Study variables include therapeutic efficacy of CQ, socio-demographic characteristics, clinical characteristics, haemoglobin concentration and parasite density.

### Early treatment failure(ETF)

Subject that has an asexual parasitaemia of any level persisting to day 7 during follow up period [6].

### Late treatment failure(LTF)

Presence of an asexual parasitaemia that becomes undetectable but reappears at any time between days 7 and 28 during follow up period [6].

### Adequate response(AR)

Absence of parasitaemia on day 28, irrespective of axillary temperature or subjects having no recurrence parasitaemia up to day 28 during follow period [6].

## Results

### Baseline characteristics of the study participants

**Fig. 1:**
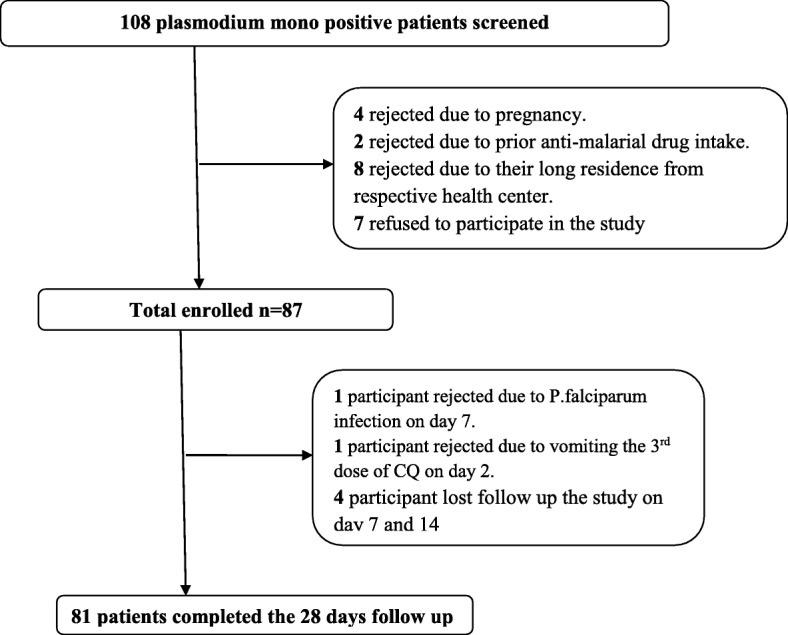
Flow chart of the study participants enrolment and follow up in the study of CQ therapeutic efficacy for treatment of *P.vivax* malaria cases in Guragea zone, southern central Ethiopia from December, 2016 - May,2017

A total of 108 *P.vivax* mono infected patients were screened in all selected health care centers, of which 87 were eligible to the 28 days follow-up study. The rest 21 participants were not enrolled in the study due to different reason. Among 87 recruited participants 81 of them had completed the entire 28 days follow up. While six were excluded because they vomited twice during the third dose of CQ, *P. falciparum* detection on day 7, and lost to follow up on day 7 and 14 as illustrated in Fig. 1.

The median age of study participants was 19 (18 months–42 years). Males were higher in proportion than females (1.2: 1). More than half of the study participants had a history of fever (57.5%) and illness two days before the day of enrolment (55.4). Geometric mean parasite density of study participants at the day of enrollment was 2270 /μl and parasitaemia clearance time for all recruited participants was observed 48 h after the day of enrollment. Mean body temperature registered on the day of recruitment was 38.2 °C and 36.8 °C on day 28. All study participants cleared fever (37.0 °C) on day 2 following treatment as shown in Table 1.

**Table 1:** Socio-demographic, parasitological and clinical characteristics of study participant recruited in the study of in vivo CQ therapeutic efficacy for treatment of *P.vivax* malaria cases in Guragea zone, southern central Ethiopia from December, 2016 - May, 2017

Variables	Total Number (%)
Gender	
Male	44(54.3)
Female	37(45.7)
Age (in years)	
Median	19
Range	1.6(18 months) - 42
Duration of illness before the day of enrollment	
One day	19(25.3)
Two days	46(55.4)
Three days	14(16.9)
Four days	2(2.4)
Average mean body temperature on day 0	38.2 °C
Average mean body temperature on day 28	36.8 °C
Mean geometric parasite density on day 0	2270 parasites /μl
Mean geometric parasite density on day 28	1390 /μl
Parasite Reduction Ratio	2.7/μl

### Efficacy outcomes

Two treatment failures (a 26 years old female on day 21 and a 28 years male on day 28) for CQ were registered. Parasitaemia persisted up to day 28 in treatment failure cases, while parasitaemia load was lower on day of admission as compared to day of enrollment (mean parasitic density on day 0 was 3700.5 parasites/μl and 139 parasites/μl on day of recurrence). PRR for treatment failure calculated was 2.7/μl (Table 1). Mean haemoglobin concentration value improvement was observed between day of enrolment (11.8 g/ dl) and day 28 (13.8 g/dl) (Table 2). Adequate response after 28 days follow up was 79 (96.0%) (Table 3).

**Table 2:** Haemoglobin concentration meseasured in the study of in vivo CQ therapeutic efficacy for treatment of *P.vivax* malaria cases in Guragea zone, southern central Ethiopia from December, 2016 - May, 2017

Anemia	Age in years (day 0)			Total	Age in years (day 28)			Total
	1–4	5–14	> 15		1–4	5–14	> 15	
Non anemia^a^	3	9	35	47	0	2	79	79
Mild^a^	4	5	18	27	0	2	0	2
Moderate^a^	3	2	2	7	0	0	0	0
Sever^a^	0	0	0	0	0	0	0	0

^a^Non anemia (> 13 g/dl), Moderate (8–10.9 g/dl), Sever (< 8 g/dl) and Mild (1112.9 g/dl)*WHO/NMH/NHD/MNM/11.1*

**Table 3:** Kaplan–Meier survival estimate of risk of therapeutic failure of CQ for the treatment of *P.vivax* malaria cases in Guragea zone, southern central Ethiopia from December, 2016 - May, 2017

Follow up period	At Risk	Excluded from the study	Treatment failure	Survival Probability Estimate	Cumulative Hazard
Day 0	87	0	0	1	0
Day 1	87	0	0	1	0
Day 2	87	1	0	1	0
Day 3	86	0	0	1	0
Day 7	86	3	0	1	0
Day 14	83	2	0	1	0
Day 21	81	0	1	0.99	0.01
Day 28	79	0	1	0.96	0.04
Total		6	2		

## Discussion

One of the greatest challenges facing malaria control today is antimalarial drug resistance. Drug resistance has been implicated in the spread of malaria to new areas and re-emergence of malaria in areas where the disease had been eradicated. Though CQ is the first-line drug for the treatment of *P.vivax* malaria in most endemic countries including Ethiopia [15], studies elsewhere [16, 17], showed increase in *P.vivax* resistance to CQ. Prevalence and geographical distribution of reported CQ treatment failures for *P.vivax* influence timely change in treatment policy in the country [8, 15].

In this in vivo CQ therapeutic efficacy study, two late parasitological treatment failures were registered. The finding is comparable with previous reports from different areas of Ethiopia; Hossana (3.3%) [18], Debrezeit 2% [19], and Serbo 3.6% [12] but when compared with previous report from Halaba special woreda in southern Ethiopia(11.7%) [20] and elsewhere [21, 22], it is lower. Variation in treatment failures observed might be due to host immunity [23], as majority of high treatment failures reported elsewhere [20–22] happened in young children which is in contrary to our results.

The changes in parasite density that occur following treatment indicate therapeutic response to anti-malarial drugs. Parasite clearance from the blood reflects the stage specificity and intrinsic potency of the anti-malarial drugs used [24, 25], in the present study parasite clearance was achieved within 48 h of drug ingestion and parasitaemia was lower on the day of recurrence than on day of enrolment for treatment failure. Similar with other studies done elsewhere [12, 18, 19], all study participants were cleared of fever following treatment. *P.vivax* is known to digest haemoglobin [26], in this study like most studies done elsewhere [12, 18, 19] mean haemoglobin improvement was also observed from day of enrolment to day 28.

Relapse, mal-absorption of drug, poor drug quality and re-infection could be factors contributing to treatment failure of *P. vivax*. Because, the drug used was confirmed to have good quality and CQ can prevent the re-infection up to 35 days, the possible causes of treatment failure in this study might be due to either mal-absorption of the drug or relapse [27]. Even though in-vivo therapeutic efficacy studies remain the ‘gold standard’ method for assessing CQ efficacy, due to the difficulty in classifying true treatment failure; as recurrences (biological resistance) or relapse due to hypnozoites stage, supplementing it with molecular test is recommended.

## Conclusions

It is important to survey and intervene CQ treatment failures before it become a public health problem. CQ remains efficacious for the treatment of *P.vivax* malaria in the study area. However, the authors believe there is a need to monitor CQ resistance by employing molecular tools for better evaluation of treatment outcomes. Despite the fact that Ethiopia is in elimination phase malaria, it is worth mentioning that prevalence of *P.vivax* observed in the study area requires strong malaria intervention measures.

## Abbreviations

CQ: Chloroquine
*P.vivax*: *Plasmodium vivax*
PRR: Parasite Reduction Ratio
